# HTLV-1 Coinfection among Patients Attending a Large HIV Treatment Centre in Trinidad

**DOI:** 10.3390/microorganisms10112207

**Published:** 2022-11-08

**Authors:** Robert Jeffrey Edwards, Karen Julien-Serrette, Jonathan Edwards, Gregory Boyce

**Affiliations:** 1 Medical Research Foundation of Trinidad and Tobago, 7 Queen’s Park East, Port of Spain, Trinidad and Tobago; 2 Department of Paraclinical Sciences, University of the West Indies, St. Augustine, Trinidad and Tobago

**Keywords:** prevalence, HTLV-1, HIV-1, coinfection, CD4+ counts

## Abstract

Studies have shown that HIV-1/HTLV-1 coinfected patients tend to have higher CD4+ counts than HIV singly infected patients. Two chart reviews were conducted at initial enrolment among patients attending a large HIV Clinic in Trinidad, one to determine the prevalence of HIV-1/HVLV-1 coinfection and another to compare the CD4+ counts and opportunistic infections among HIV-1/HTLV-1 coinfected patients compared to a randomly selected comparison group of HIV-1 singly infected patients. Sociodemographic, clinical and laboratory data were collected and analysed using SPSS Version 25. During the period April 2002–December 2018, 8916 HIV-1 patients were enrolled at the clinic; 159 were HIV-1/HTLV-1 coinfected; the age range was 18–81 years; the median age was 40 years; 87 (54.7%) were females; and the median CD4+ count and median HIV-1 viral load at enrolment were 300 cells/mm^3^ and 128,543 copies/mL, respectively, with an HTLV-1 seroprevalence of 1.78%. Among the 477 HIV-1 singly infected patients, the age range was 18–71 years; the median age was 33 years; 248 (52.0%) were males; and the median CD4+ count and the median HIV viral load were 295 cells/mm^3^ and 23,369 copies/mL, respectively. Opportunistic infections (OIs) were diagnosed in 59 (37.1%) of the coinfected patients versus 48 (10.1%) among those HIV singly infected (*p* < 0.001). HIV-1/HTLV-1 coinfected patients had higher HIV-1 viral loads (*p* < 0.001) and more OIs, suggesting a worse prognosis though there were no statistically significant differences in CD4+ counts (*p* = 0.96) as compared to the HIV-1 mono-infected patients.

## 1. Introduction

HTLV-1 was the first retrovirus to be identified by Poiesz et al. (1) in 1980 when HTLV-1 was isolated in a cell line from a patient with cutaneous T-Cell lymphoma [1] and HTLV-1 was endemic in southeast Japan, sub-Saharan Africa, the Caribbean, South America, central parts of Australia, Melanesia and the Middle East [2,3]. HTLV-1 infection is considered a neglected disease and generally not afforded priority by public health authorities, and silent transmission occurs as an early infection is usually asymptomatic, and disease manifestations present later in life [4].

The most important routes of transmission are mother to child, mostly through prolonged breastfeeding [5,6]; sexual intercourse (male to female more efficient) [7]; and parenterally via blood and blood products and sharing of needles [2]. HTLV-1 mainly infects the CD4+ T cells via cell-to-cell interactions [8]. The seroprevalence of HTLV-1 is age and sex-dependent; it increases with age and is higher in females [9].

It is estimated that around 5–10 million people globally may be infected with HTLV-1 [3], and about 95% of patients infected with HTLV-1 remain asymptomatic [3]. HTLV-1-associated diseases include adult T-cell leukemia/lymphoma (ATLL) [1,10], tropical spastic paraparesis/HTLV-1 associated myelopathy (TSP/HAM) [3,11], infective dermatitis [12] and uveitis [13].

The twin island republic of Trinidad and Tobago (T&T), with a population of approximately 1,399,491 persons (2020 mid-year estimate), are the southernmost islands of the Caribbean. The multi-ethnic population is comprised of persons of East Indian origin (35.4%), persons of African descent (34.2%), those of mixed race (23.8%) and 8.4% of other ethnic groups (Asian, European, Middle Eastern). Persons of African ancestry came to the Caribbean via the African slave trade as plantation workers, mainly from West and Central Africa [14,15,16], where HTLV-1 is endemic. After the abolition of slavery, the East Indians came to Trinidad as indentured labourers [17], mainly from Bengal, Bihar and Utter Pradesh, where the prevalence of HTLV-1 is very low [18], and this may support the hypothesis that the African slave trade may be responsible for endemicity of HTLV-1 in the Caribbean [19].

In 1986, 1558 serum samples stored at −20 °C from a 1982 population-based hepatitis serosurvey in Trinidad were screened for HTLV-1 antibodies [20]. The HTLV-1 seroprevalence in the total sample was 2.2% (34/1558); among persons of African origin, the HTLV-1 seroprevalence was 3.2% (33/1035) and 0.2% (1/487) among persons of east Indian origin [20], showing a racial disparity.

The first cases of AIDS in Trinidad and Tobago were reported in 1983 [21] among men who have sex with men (MSM), with a transition to mainly heterosexual transmission of HIV in 1985 [22]. Antiretroviral therapy (ART) became available free of charge to persons living with HIV (PLHIV) in 2002 [23] supported by the government [24], and it is estimated that there are 10,000 PLHIV in Trinidad and Tobago and approximately 70% of these persons are on ART [25].

Studies have shown that HTLV-1/HIV-1 coinfected individuals may have higher CD4+ lymphocyte counts than those singly infected with HIV-1 [26]. It is thought that the higher CD4+ counts in some coinfected individuals may suggest HTLV-I-associated nonspecific lymphocyte proliferation and clonal expansion of the CD4+ infected lymphocytes. The elevated CD4+ cell count in some coinfected individuals may not necessarily provide immunological benefit as most of the cells may be functionally impaired and may not be a reflection of a competent immune system [26].

The purpose of the study was to determine the prevalence of HTLV-1 infection in patients attending the HIV Clinic and to compare the CD4+ counts and opportunistic infections of HIV-1/HTVLV-1 coinfected patients versus HIV-1 singly infected patients at the initial visit to the HIV Clinic. 

## 2. Methodology

The Medical Research Foundation of Trinidad and Tobago (MRFTT) is the largest HIV Treatment and Care Centre in the English-speaking Caribbean, and as of December 2018, there were 8916 HIV-infected patients registered. As part of the routine clinical care, all patients attending the HIV Clinic on their first visit are screened for HTLV-1 antibodies using an enzyme-linked immunosorbent assay (ELISA). Until 2012, the Vironostika HTLV-1 ELISA (Vironostika, Organon Teknika, Durham DC) was used as the screening ELISA, which was then discontinued and followed by the use of the HTLV-1/2 Ab Diapro ELISA (Dia.Pro, Sesto San Giovanni, Italy). In 2018, all patients with HTLV-1 positive ELISA results were re-screened with the HTLV-1/2 Ab Diapro ELISA (Dia.Pro, Italy), and all positive samples were confirmed by Western blot (MP Diagnostic ^TM^ HTLV BLOT 2.4 Western Blot Assay, MP Biomedicals Asia Pacific Pte. Ltd., Singapore), which can differentiate antibodies to HTLV-1 and HTLV-2. Testing for HIV-1 was performed using Alere Determine^®^ HIV-1/2 Ab rapid test (Alere Inc., Waltham, MA, USA) and the Uni-Gold^®^ Recombigen HIV-1/2 Ab rapid test (Trinity Biotech PLC, Bray, Ireland), and all positive samples were confirmed by enzyme-linked immunosorbent assay (ELISA) testing in accordance with the National HIV Testing Algorithm.

The MRFTT has an electronic medical records system named CELLMA. A list of HIV-1/HTLV-1 coinfected patients who ever attended the clinic during the period April 2002–December 2018 was generated using CELLMA, and a chart review was conducted.

### 2.1. Inclusion Criteria

1. Age 18 years and older;
2. Patients living with HIV;
3. Patients attending the MRFTT and HIV-1/HTLV-1 coinfected.

### 2.2. Exclusion Criteria

1. Age under 18 years old;
2. HIV-1 seronegative individuals;
3. Patients attending the MRFTT who are not HTLV-1 coinfected.

A randomly selected comparison group of HIV-1 singly infected patients enrolled at the clinic over the same period (April 2002–December 2018) in a ratio of 3:1 (singly HIV infected: coinfected) was drawn from the entire population of HIV-1 singly infected patients at the clinic via computer-generated random numbers using their unique clinic identification numbers. The randomised list of HIV-1 singly infected patients was then input into CELLMA, and a chart review on these patients was carried out.

### 2.3. Ethical Approval

The study and related study procedures were reviewed and approved by the Campus Research Ethics Committee, University of the West Indies, St Augustine, Trinidad, approval number CEC444/02/18.

### 2.4. Data Analysis

Data were abstracted from client records to obtain the sociodemographic, clinical records and laboratory diagnosis of HTLV-1. SPSS Version 25 was utilised for statistical analysis. The prevalence of HIV-1/HTLV-1 coinfection was determined by dividing the number of patients who had been given a diagnosis of HIV-1/HTLV-1 coinfection by the number of people in the clinic population during the study time period. Any missing data were dropped from the statistical analysis. Stratified, simple random and quota sampling methods were used to select the HIV-1 singly infected sample population. Data were analysed using Measures of Central Tendency (mean, median), bivariate analysis, Chi-square tests (X^2^), Fisher’s Exact Test and the Mann–Whitney U Test (Wilcoxon Rank Sum Test). The dataset comprised categorical variables, such as type (HIV-1 vs. HIV-1/HTLV-1), ethnicity, sex, sexual orientation (heterosexual vs. homosexual/bisexual), opportunistic infections, AIDS-defining infections and current status, and numerical variables, such as age, CD4+ count and HIV-1 viral load. A *p*-value < 0.05 was considered statistically significant.

## 3. Results

During the period April 2002–December 2018, there were 8916 patients registered at the HIV MRFTT Clinic: 6565 (73.6%) were patients in active care and followed up; 1603 (18.0%) had died; 418 (4.7%) were lost to follow-up; and 330 (3.7%) were transferred to another clinic. The ethnic disposition of persons attending the clinic consisted of 5322 (59.7%) persons of African descent, 692 (7.8%) persons of East Indian origin, 2721 (30.5%) persons of mixed race and 67 (0.75%) other persons (Chinese, Caucasian, Syrian/Lebanese). Data on ethnicity were not available for 114 (1.3%) persons.

(1) One hundred and fifty-nine HIV-1/HTLV-1 coinfected clients were seen: 72 were males (45.3%); 87 were females (54.7%); the median age was 40 years at the initial visit; and the median nadir CD4+ count was 300 cells/mm^3^ (Table 1). No HIV-1/HTLV-2 coinfected cases were diagnosed.

The HTLV-1 seroprevalence in the HIV Clinic at MRF:

- Entire HIV Clinic Population (159/8916) = 1.78%;
- Persons of African origin (132/5322) = 2.48%;
- Persons of East Indian origin (6/692) = 0.87%;
- Persons of Mixed race (21/2721) = 0.77%.

A randomly selected comparison group of 477 HIV-1 singly infected patients enrolled at the clinic over the same period (April 2002–December 2018) was chosen, with an age range of 18–71 years, and the median age was 33 years at the initial visit. There were 248 (52.0%) males and 229 (48.0%) females, and the median (nadir) CD4+ count was 295 cells/mm^3^ (Table 1). At each person’s initial visit, all study patients were treatment naïve, allowing for between-group comparisons of HIV-1 viral load, CD4 counts, opportunistic infections and AIDS-defining opportunistic infections.

“Treat all”, where all HIV-infected patients are offered ART irrespective of CD4+ T-cell count, was instituted in T&T in 2017. The status of the HIV-1/HTLV-1 coinfected patients at the HIV MRFTT Clinic as of 31 December 2018 in Table 1 showed that 139 (87.4%) of the patients were in active follow-up, and of these, 138 (99.3%) were on ART, 12 (7.6%) defaulted from the clinic, and there were 8 (5.0%) deaths—7 (87.5%) deaths with AIDS-related complications and 1 (12.5%) died from complications of alcohol-related liver disease. This was compared to the status of the 477 HIV-1 singly infected patients on 31 December 2018, which showed that 333 (69.8%) of the patients were in active follow-up (*p* < 0.001), and of these, 328 (98.5%) were on ART (*p* = 0.98), 63 (13.2%) defaulted from the clinic (*p* = 0.02), and there were 81 (17.0%) deaths (*p* < 0.001). Of the 81 deaths, 48 (59.1%) were associated with AIDS-related complications, 18 (22.2%) deaths were due to complications of chronic noncommunicable diseases (e.g., diabetes mellitus, hypertension, cardiovascular disease, etc.), cancer and traumatic injuries (road traffic accidents, falls, violence, etc.), and 15 (18.5%) deaths were unknown/not documented in the patient files. As of December 31, 2018, the median CD4+ counts and median HIV-1 viral loads among the HIV-1/HTLV-1 coinfected patients were 673 cells/mm^3^ and <40 copies/mL, respectively, and among the HIV-1 singly infected were 568 cells/mm^3^ and <40 copies/mL, respectively.

Statistically significant findings in HIV-1/HTLV-1 coinfected patients at the initial visit as compared to the randomly selected HIV-1 singly infected group included older median age (*p* < 0.001), higher median HIV-1 viral loads (*p* < 0.001), being diagnosed with an opportunistic infection (*p* < 0.001), being diagnosed with an AIDS-defining illness (*p* < 0.001), African ethnicity (*p* < 0.001) and heterosexual sexual orientation (*p* = 0.003). However, there were no statistically significant associations between females and males (*p* = 0.10) between the two groups of patients.

Among the 159 HIV-1/HTLV-1 coinfected patients, at initial presentation (Table 2), there were 59 (37.1%) patients (37 males and 22 females) with an opportunistic infection, and 38 (23.9%) of these (22 among males and 16 among females) were AIDS-defining illnesses diagnosed as compared to 48 (10.1%) patients (19 females and 29 males) with opportunistic infections (*p* < 0.001), and 28 (5.9%) of these (12 among females and 16 among males) were AIDS-defining illnesses (*p* < 0.001) diagnosed at initial presentation among the 477 HIV-1 singly infected patients.

In the HTLV-1/HIV coinfected patients, 38.8% had CD4+ counts <200 cells/mm^3^, 71.7% had CD4+ counts <500 cells/mm^3^ and 28.3% had CD4 > 500 cells/mm^3^ at the initial clinic visit as compared to the HIV-1 singly infected patients where 33.3% had CD4+ counts <200 cells/mm^3^, 76.9% had CD4+ counts <500 cells/mm^3^ and 23.1% had CD4 > 500 cells/mm^3^. However, there were no statistically significant differences among the median CD4+ counts between both groups of patients.

The HTLV-1-associated conditions in the 159 HIV-1/HTLV-1 coinfected patients attending the HIV Clinic included Tropical Spastic Paraparesis (TSP) 4.4% (7/159), HTLV-1 associated lymphoma 0.63% (1/159) and infective dermatitis 0.63% (1/159).

## 4. Discussion

The HTLV-1 prevalence in HIV-1 infected patients attending the Clinic at the MRFTT was 1.78%, with a higher seroprevalence in patients of African origin (2.48%) compared to those of East Indian origin (0.87%) and in the HIV-1/HTLV-1 coinfected study patients, there were 132 (83.0%) persons of African origin as compared to 286 (68.5%) of the HIV-1 singly infected patients (*p* < 0.001). These results are similar to that obtained by Blattner et al. in 1986 [14] in a general population sample in Trinidad, where the HTLV-1 seroprevalence was 3.2% among persons of African origin and 0.2% among those of East Indian origin and lent credence to the theory that HTLV-1 may have come to the Caribbean via the African slave trade [20].

HIV-1/HTVLV-1 coinfected patients at the initial visit had a median CD4+ of 300 cells/mm^3^, which was similar compared to a median CD4+ of 295 cells/mm^3^ in HIV-1 singly infected patients at enrolment, but this was not statistically significant (*p* = 0.96). This was in contrast to the study by Schechter et al. [26], where HIV-1/HTLV-1 coinfected persons had approximately 78% higher CD4+ counts compared to those singly infected with HIV-1 [26], and the study by Gudo et al. [27], where the CD4+ counts were significantly higher in the coinfected patients [27]. In addition, studies showed that HIV-1/HTLV-1 coinfected patients maintain stable CD4+ T-cell counts that may conceal the immunosuppression associated with the progression to AIDS, which may affect the clinical decision-making related to prophylaxis for opportunistic infections [26,27]. HTLV-1 promotes the clonal expansion of infected CD4+ lymphocytes, which may result in artificially elevated CD4+ counts in some HIV-1/HTLV-1 coinfected persons [26]. The elevated CD4+ counts in coinfected patients may not necessarily suggest a competent immune system, as most of the cells tend to be functionally impaired [26,27].

At the initial visit, the HIV-1/HTLV-1 coinfected patients had a median viral load of 128,543 copies/mL, 59 (37.1%) were diagnosed with opportunistic infections, and 38 (23.9%) of these were AIDS-defining illnesses. In comparison, HIV-1 singly infected patients had a median HIV-1 viral load of 23,369 copies/mL (*p* < 0.001), 48 (10.1%) were diagnosed with opportunistic infections (*p* < 0.001) and 28 (5.9%) were diagnosed with AIDS-defining illnesses (*p* < 0.001). Thus, the HIV-1/HTLV-1 coinfected patients had higher HIV-1 viral loads and were more symptomatic, suggesting a worse prognosis as compared to the HIV-1 singly infected patients. Studies show that the natural history of HIV-1 is modified by HIV-1/HTLV-1 coinfection with accelerated progression to AIDS, worse outcomes of HIV-related opportunistic infections and a shorter survival time [26,28]. A systematic review comparing the clinical and laboratory outcomes in HIV-1/HTLV-1 coinfection versus HIV-1 singly infected patients concluded that coinfected patients had higher CD4+ cell counts, shorter survival and increased progression to death, hence higher mortality than HIV-1 mono-infected patients [29]. It has been suggested that HIV-1/HTLV-1 coinfection may be associated with chronic immune activation and immune dysregulation with an increase in dysfunctional CD4+ T-lymphocytes as a result of the lymphoproliferative effect of HTLV-1 [27,29,30,31] and faster clinical progression. However, this is controversial as some studies proposed that HIV-1/HTVLV-1 coinfection may result in delayed HIV progression to AIDS and does not seem to result in shortened survival [32,33]. In our study, as of 31 December 2018, there were 8 (5.0%) deaths in the HIV-1/HTLV-1 coinfected patients as compared to 81 (17.0%) deaths in the HIV singly infected patients (*p* < 0.001). The decreased deaths among the HIV-1/HTLV-1 coinfected patients in the study may be due to the criteria for eligibility for antiretroviral therapy (ART) at the HIV clinic, as before 2017, all HTLV-1/HIV-1 coinfected patients were offered ART irrespective of CD4+ cell count; however, HIV-1 singly infected patients were only offered ART if they had CD4+ counts less than 350 cells/mm^3^. These results are supported by a study conducted by Collins et al. [34] at an HIV reference centre in Peru, which showed that HIV-1/HTLV-1 coinfection was not associated with an increased risk of death, increased risk of mortality in this population was associated with the presence of AIDS-defining illnesses and the absence of ART [34]. However, “treat all” was instituted in 2017 in T&T, and on 31 December 2018, of the patients in active care and follow-up, 99.3% of the HIV-1/HTLV-1 coinfected and 98.5% of the HIV-1 singly infected patients were on ART. Studies have shown that combination ART normalises survival time in coinfected patients [35].

In our study, among the HIV-1/HTVLV-1 coinfected patients, the median age was 40 years, there were 87 (54.7%) females and 148 (93.4%) self-identified as heterosexual sexual orientation. In comparison, among the HIV singly infected patients at the initial visit, the median age was 33 years (*p* < 0.001), there were 229 (48.0%) females (*p* = 0.10) and 412 (86.4%) self-identified as heterosexual (*p* = 0.004). Studies have shown that sexual transmission of HTLV-1 tends to occur mainly (but not exclusively) from male to female [3,9], and in our study, there were more females (54.7%) in the coinfected group as compared to the HIV singly infected group (48.0%) (*p* = 0.10). Though not statistically significant, this supports studies that show the increased HTLV-1 seroprevalence with age among women [3,9] and may account for the higher frequency of heterosexual sexual orientation among co-infected versus HIV singly infected persons in the study. Due to high-risk behaviours, MSM may be at high risk of acquiring HTLV-1; however, a study in Central Brazil conducted among 430 MSM showed an HTLV-1 seroprevalence of 0.7%, which was similar to the HTLV-1 prevalence of 0.48% among blood donors [36].

In our study, the HTLV-1-related diseases seen in our cohort of HIV-1/HTLV-1 coinfected patients included Tropical Spastic Paraparesis (TSP)/HTLV-1 myelopathy (HAM) 4.4% (7/159), HTLV-1 associated lymphoma 0.63% (1/159) and infective dermatitis 0.63% (1/159). The patient with infective dermatitis was diagnosed in childhood and, as a teenager, acquired HIV-1 by sexual transmission. Studies have suggested that of the HTLV-1-associated diseases, higher rates of myelopathy (TSP/HAM) were seen in HIV-1/HTLV-1 coinfected patients [29,37,38], which was shown in our study, as in addition to immune dysfunction, both viruses are neuropathogenic, which may result in neurological diseases via direct and indirect mechanisms [39].

Though our study includes the largest number of HIV-1/HTLV-1coinfeted patients in the English-speaking Caribbean, there are a number of limitations. One major limitation of the study is the uncertainty of the duration of HTLV-1 infection in the HIV-1/HTLV-1 coinfected patients. Before ART became available in Trinidad, Blattner et al. [40] followed a cohort of patients with acute HIV-1 infection and showed that the time to progression to AIDS was 4.8 years and the time to death was 5.6 years [40]. On the basis of this and the older age of the HTLV-1 co-infected patients, it may be postulated that HTLV-1 may have preceded the HIV-1 infection. It has been reported that there was a late presentation to care among HIV/AIDS patients in the Caribbean earlier in the HIV epidemic [41], possibly due to less widespread availability of HIV testing and anti-retroviral therapy. However, 38.8% and 33.3%, respectively, of the HTLV-1/HIV-1 coinfected patients and HIV-1 mono-infected patients, respectively, had nadir CD4+ counts <200 cells/mm^3^, indicating similar late presentation to care in both groups of patients.

## 5. Conclusions

This is the largest study in the Caribbean documenting the prevalence of HTLV-1 coinfection among patients attending an HIV Clinic. The HTLV-1 seroprevalence was 1.78% and was higher in persons of African origin; thus, HTLV-1 testing should be conducted among HIV-1-infected patients from the Caribbean. Other studies have shown that HIV-1/HTLV-1 coinfected patients may have artificially elevated CD4+ counts, though, in this study, there was no statistically significant difference in CD4+ counts between HIV-1/HTLV-1 co-infected and HIV-1 singly infected patients; however, the coinfected patients had higher HIV-1 viral loads and more opportunistic infections suggesting a worse prognosis as compared to the HIV singly infected patients at the initial visit.

## Figures and Tables

**Table 1:** Baseline characteristics of the study population at initial visit (2002–2018).

Descriptive Statistic	HIV-1/HTLV-1 Coinfection Total (*n* = 159)	Randomly Selected HIV-1 Singly Infected Comparator Group (*n* = 477)	*p* Value
Median Age	40 years	33 years	<0.001
Age Range	18–81 years	18–71 years	
Sex			
Males Females	72 (45.3%) 87 (54.7%)	248 (52.0%) 229 (48.0%)	0.06 0.10
Ethnicity		(*n* = 417)	
African	132 (83.0%)	286 (68.5%)	<0.001
East Indian	6 (3.8%)	49 (11.8%)	<0.001
Mixed	21 (13.2%)	77 (18.5%)	0.14
Other (Caucasian)		5 (1.2%)	
Current status (Dec 2018)			
Alive Deceased Defaulted from Clinic	139 (87.4%) 8 (5.0%) 12 (7.6%)	333 (69.8%) 81 (17.0%) 63 (13.2%)	<0.001 <0.001 0.02
Sexual Orientation	(*n* = 157)		
Heterosexual Homosexual/Bisexual	148 (94.3%) 9 (5.7%)	412 (86.4%) 65 (13.6%)	0.004 0.003
Opportunistic infections	59 (37.1%)	48 (10.1%)	<0.001
AIDS-defining illnesses	38 (23.9%)	28 (5.9%)	<0.001
Medan (nadir) CD4+ count (cells/mm^3^)	(*n* = 152)		
	300	295	0.96
Median HIV-1 viral load (copies/mL)	128,543	23,369	<0.001
Patients on ART (December 2018)	(*n* = 139)	(*n* = 333)	
	138 (99.3%)	328 (98.5%)	0.98

**Table 2:** Comparison of opportunistic infections at initial visit 2002–2018 and deaths on 31 December 2018 in HIV-1/HTLV-1 coinfected patients versus randomly selected HIV singly infected patients.

Opportunistic Infection/Deaths	HIV-1/HTLV-1 Coinfection Total (*n* = 159)	Randomly Selected HIV-1 Singly Infected Comparator Group (*n* = 477)	*p* Values
Opportunistic infections			
Oral candidiasis	11 (6.9%)	12 (2.5%)	
Oral candidiasis and Pruritic papular eruption	8 (5.0%)	5 (1.0%)	
Oral candidiasis and herpes zoster	2 (1.3%)	3 (0.6%)	
AIDS-defining illnesses			
Esophageal candidiasis	2 (1.3%)	4 (0.8%)	
HIV wasting syndrome	7 (4.4%)	6 (1.3%)	
Pneumocystis pneumonia (PCP)	7 (4.4%)	4 (0.8%)	
Tuberculosis- pulmonary/extra-pulmonary	7 (4.4%)	9 (1.9%)	
Cerebral toxoplasmosis	6 (3.8%)	3 (0.6%)	
Cryptococcal meningitis	2 (1.3%)		
Cervical cancer	2 (1.3%)		
Cytomegalovirus (CMV) retinitis	1 (0.6%)		
Cutaneous/disseminated histoplasmosis	1 (0.6%)		
PCP and cerebral toxoplasmosis	1 (0.6%)		
PCP and disseminated histoplasmosis		1 (0.2%)	
PCP and esophageal candidiasis	1 (0.6%)	1 (0.2%)	
CMV retinitis and TB	1 (0.6%)		
Total Opportunistic infections	59 (37.1%)	48 (10.1%)	<0.001
Deaths			
AIDS-related complications	7 (4.4%)	48 (10.1%)	
Other causes	1 (0.6%)	18 (3.8%)	
Unknown/not documented		15 (3.1%)	
Total Deaths	8 (5.0%)	81 (17.0%)	<0.001

## Data Availability

All data analysed or generated during this study are included in the manuscript.
